# Molecular and metabolic insights into floral scent biosynthesis during flowering in *Dendrobium chrysotoxum*

**DOI:** 10.3389/fpls.2022.1030492

**Published:** 2022-11-28

**Authors:** Zhihui Du, Yuxuan Jin, Weize Wang, Kuaifei Xia, Zhilin Chen

**Affiliations:** ^1^ Guizhou Horticulture Institute, Guizhou Academy of Agricultural Sciences, Guiyang, China; ^2^ Key Laboratory of South China Agricultural Plant Molecular Analysis and Genetic Improvement and Guangdong Provincial Key Laboratory of Applied Botany, South China Botanical Garden, Chinese Academy of Sciences, Guangzhou, China

**Keywords:** *Dendrobium chrysotoxum*, floral scent, widely-targeted volatilomics, terpenes, RNA-Seq

## Abstract

*Dendrobium chrysotoxum* is considered as an important ornamental dendrobium because of its strong and long-lasting floral scent. Nevertheless, few information is known about the dynamic changes and related formation mechanism of dendrobium floral scent at different flowering stages. In this study, the characteristics and biosynthetic mechanism of floral scent in *D. chrysotoxum* during flowering was revealed by using widely-targeted volatilomics (WTV) combined with transcriptome analysis. Over 500 kinds of volatile organic compounds (VOCs) were detected in the floral scents of *D. chrysotoxum*, which improved the knowledge about floral scent components of dendrobium. A total of 153 differential VOCs and 4,487 differentially expressed genes (DEGs) were identified between flowers of different flowering stages, respectively. The results for both volatilomics and transcriptomics data indicated that terpenes and related genes played an important role in the formation of floral characteristics of *D. chrysotoxum*. But in general, the expression of genes showed an opposite trend to the accumulation of metabolites during flowering, suggesting that the regulation of floral scent biosynthesis might have started at the budding stage in *D. chrysotoxum*. Additionally, a transcriptional metabolic regulatory network consisting of terpenes, terpene synthases and candidate transcription factors was established. This research is the first systematic and comprehensive exploration of floral characteristics and related mechanisms during flowering in *D. chrysotoxum*. It provides basis for exploration of mechanisms on the floral scents and the breeding of aromatic dendrobium.

## Introduction

*Dendrobium chrysotoxum* Lindl is an epiphytic perennial herb of the Orchidaceae family. It is widely distributed across Southwestern China, Northeastern India and Southeast Asian countries such as Myanmar, Laos, and Thailand (Takamiya et al., 2014; Chase et al., 2015). *D. chrysotoxum* has high medicinal value due to its enrichment of metabolites such as alkaloids, polysaccharides, and polyphenols in its stem (Shang et al., 2021; Liu et al., 2022). Additionally, due to the pure bright yellow color and strong fragrance in its flowers, *D. chrysotoxum* has ornamental values as great as potted and cut flowers (Robustelli della Cuna et al., 2021).

Floral scent is one of the most significant traits for evaluating the ornamental value of flowering plants. Several studies have found that consumers were more affected by floral scent than flower color or shape (Dudareva et al., 2013; Fan et al., 2019). Nevertheless, scent was difficult to observe because its complexity, variability and invisibility. Due to these characteristics, there has been few researches on dendrobium floral scent, as compared to flowering period, color and shape (Wang et al., 2022). From the standpoint of floral characteristics, *D. chrysotoxum* was regarded as a precious parent for the breeding of aromatic dendrobium owing to its strong floral scent and longer flowering period. Meanwhile, some candidate genes involved in aromatic metabolic pathways have been identified in D. chrysotoxum (Zhang et al., 2021). Based on these advantages, *D. chrysotoxum* could be selected as an ideal specie for study of dendrobium floral scent.

‘Floral scent’ is a collective term for all volatile metabolites released by plant flowers, which is usually composed of an array of volatile organic compounds (VOCs) with low molecular mass, such as terpenoids, benzenoids/phenylpropanoids, and aliphatics (Knudsen et al., 2006; Dudareva et al., 2013). Over the years, there were few reports about the investigation of the components and formation mechanism of dendrobium floral scent, compared with common aromatic plants, like rose and lily (Hendel-Rahmanim et al., 2007; Du et al., 2019). Thanks to the change in flower market preference and breeding direction, some researches focused on aromatic compounds of dendrobium have been reported in recent years, the floral components of several representative aromatic dendrobium species were preliminarily analyzed. For the first time, Huang et al. determined the floral scent of fresh flowers of *D. chrysotoxum* and found that the ester and terpenoids were the dominant components, their content exceeded 80% of all detected compounds. Octyl acetate, β-ocimene, α-pinene and hyacinthin were likely to be the main substances responsible for its floral scent formation (Huang et al., 2018). 41 VOCs were detected in the floral compounds of *D. moniliforme*, and α-pinene was considered by researchers to be the main component of its floral fragrance (Qiu et al., 2019). Subsequently, 72 VOCs in the flowers of two other aromatic *Dendrobium* species (*D. hancockii* and *D. trigonopus*) were examined, results showed that the main volatile constituent in both the two species was terpenoids, including β-ocimene, β-caryophyllene, linalool and limonene (Wang et al., 2020). These findings not only suggested the diversity and complexity of the volatile components in flowers of *Dendrobium* species, but also provided some theoretical reference for its utilization in perfumes and cosmetics.

With the development of molecular biological techniques, high-throughput sequencing was gradually used to study the biosynthetic mechanism of the floral aroma of dendrobium. Several biosynthetic pathways of fragrance compounds and some structural genes encoding key enzymes have been demonstrated in *D. officinal*, which was the first species to complete whole genome sequencing in *Dendrobium* genus (Zhao et al., 2020; Li et al., 2021). However, *D. officinal* was not outstanding in terms of floral scent, it was not the ideal material for studying the mechanism of floral scent in dendrobium. In addition, the emission of floral scent was dynamic with the changes of flowering period (Dudareva et al., 2013). Therefore, when conducting on floral scent, it was critical to take the associated release dynamics into account (Fan et al., 2019). Nevertheless, the effect of flowering period was ignored in existing studies on the mechanism of dendrobium floral scent.

To reveal the dynamic characteristic and related mechanisms of floral scent in dendrobium, a series of floral organs of *D. chrysotoxum* at different flowering stages were analyzed using widely-targeted volatilomics (WTV) and RNA-seq analysis. The comparative analysis was performed to identify the differential volatile organic compounds (DVOCs) and differentially expressed genes (DEGs). Finally, the combined analysis between metabolites and genes data supplied deeper understanding of floral scent properties and candidate regulatory genes in *D. chrysotoxum*. This work provided new insights into the dynamic release and regulatory mechanisms of floral scent in dendrobium.

## Materials and methods

### Plant materials and RNA extraction

**Figure 1:**
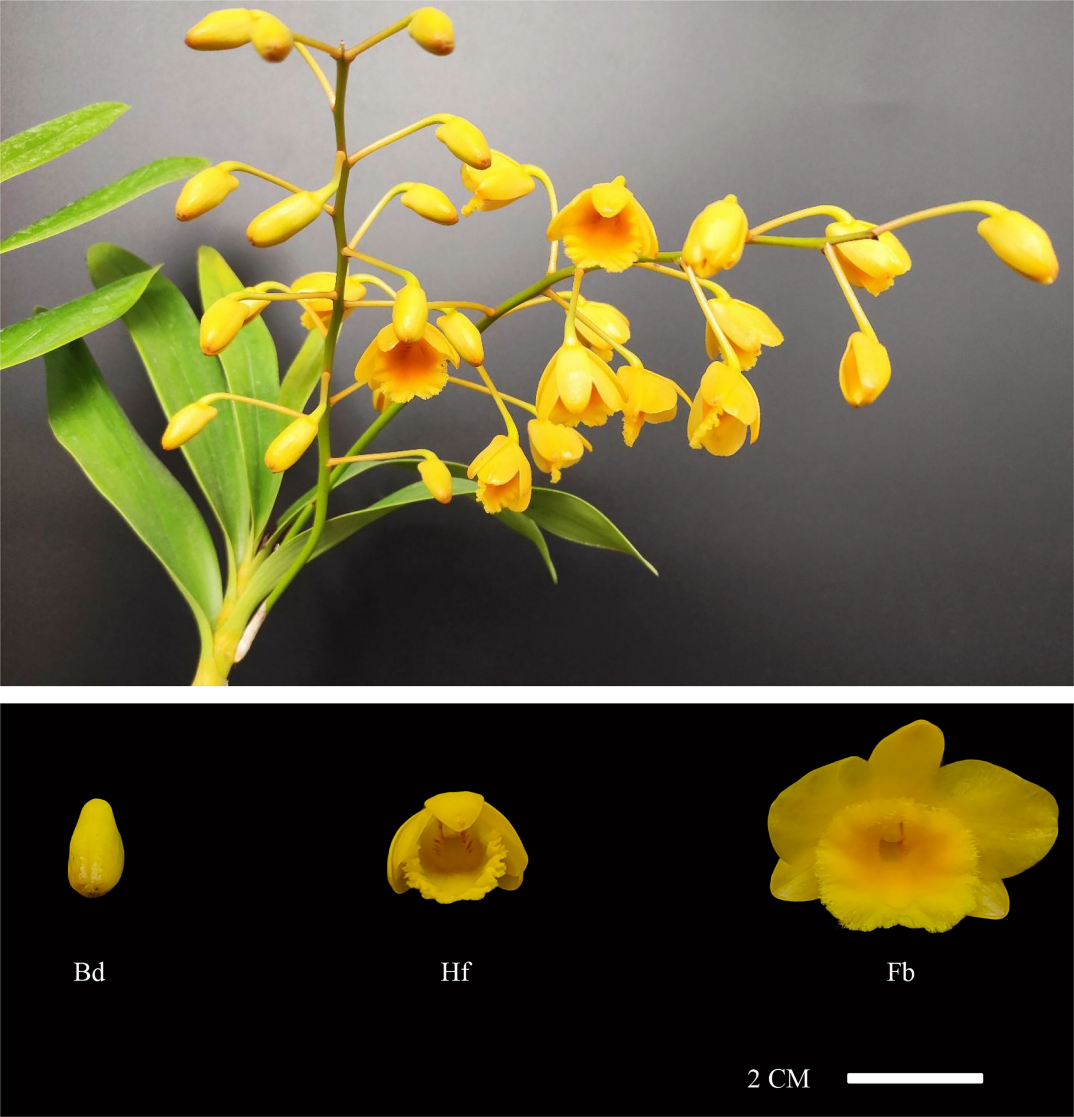
Floral organs phenotypes of *D. chrysotoxum* at different flowering stages. Bd, budding stage; Hf, half-open stage; Fb, full-bloom stage.

*D. chrysotoxum*, originated from Yunan province (China), were collected and cultivated at the Institute of Horticulture, Guizhou Academy of Agricultural Sciences (Guiyang, China). To investigate the biosynthetic mechanism of floral scent composition during flowering process in *D. chrysotoxum*, a series of samples from its floral organs at different flowering stages were collected, including the budding stage, the half-open flowering stage and the full-bloom flowering stage (**Figure 1**). To avoid the influence of environmental factors as much as possible, sampling was carried out uniformly at 3 days after the opening of the first bloom at the base of the inflorescence. At this time, the bottom blooms of the same inflorescence showed full-bloom state, the middle blooms showed half-open state and the top blooms showed closed state. All samples from three flowering stages were collected and immediately frozen in liquid nitrogen, then stored at -80°C until needed. Each group of samples consisted of nine biological replicates. Of which, six replicates were used for volatile metabolomics determination and three replicates were used for RNA extraction, respectively.

Total RNA of each sample was extracted using Trizol (Invitrogen, MA, USA) in terms of the manufacturer’s protocol. The integrity of RNA was monitored by 1.0% agarose gel electrophoresis and RNA 6000 Nano Assay Kit of the Bioanalyzer 2100 system (Agilent Technologies, CA, USA). And the Qubit^®^2.0 Flurometer (Life Technologies, CA, USA) was used to measure the concentration of RNA.

### Widely-targeted volatilomics and analysis

The strategy of widely-targeted volatilomics (WTV) referred to the published research by Hainan University (Yuan et al., 2022). In brief, head-space solid-phase microextraction (HS-SPME) was employed to collect the volatiles from flower tissues, which were absorbed by a 120 µm divinylbenzene/carboxen/polydimethylsiloxane fiber (Agilent Technologies, CA, USA) for 15 min at 100 °C. Then, the collected volatiles were detected by gas chromatography-mass spectrometer (GC-MS) using an Agilent 8890 GC and Agilent 5977B MS, which equipped with a DB-5MS capillary column (30 m × 0.25 mm × 0.25 μm; Agilent Technologies) and helium was used as the carrier gas at a linear velocity of 1.2 mL/min. The temperature of the injector was kept at 250°C. The oven temperature was programmed as follows: 40°C for 3.5 min, increasing at 10°C/min to 100°C, followed by an increase at 7°C/min to 180°C, then increasing at 25°C/min to 280°C with hold for 5 min. The temperatures of quadrupole mass detector, ion source and transfer line were set at 150, 230 and 280°C, respectively. The electron ionization potential of the mass spectra was 70 eV, and selected ion monitoring (SIM) mode was used for the identification and quantification of analytes.

The identification of volatile compounds was achieved by comparing the mass spectra with the database (NIST20 and MWGCSIM1). Qualitative analysis of the raw data obtained was performed using Qualitative Analysis Workflows B.08.00, and quantitative analysis was performed using masshunter. The internal standard was used to normalize the quantitative data. Subsequently, the volatilomics data were analyzed using the OPLS-DA (orthogonal partial least-squares discrimination analysis) model. VIP (variable importance for projection) values of metabolites were extracted from result of OPLS-DA. Differential volatile organic compounds (DVOCs) between groups were determined by VIP (VIP ≥ 1) and Log_2_ fold change (|Log_2_ fold change | ≥ 1.0).

### RNA-seq and transcriptome data analysis

Sequencing libraries were constructed using NEBNext^®^ UltraTM RNA Library Prep Kit for Illumina^®^ (NEB, Ipswich, MA, USA) following the manufacturer’s protocol. An AMPure XP system (Beckman Coulter, Beverly, MA, USA) was used to screen 250–300 bp cDNA, and polymerase chain reaction (PCR) amplification was performed. RNA-Seq was performed by MetWare (Wuhan, China) on the Illumina NovaSeq platform, and 125 bp paired-end reads were generated.

Adapters and low-quality (sequences with N base content exceeding 10% or Q ≤ 20 base content exceeding 50%) sequences of raw reads were trimmed by fastp (Chen et al., 2018). Using hisat2 to build the index of reference genome of *D. chrysotoxum* (NCBI accession number: GCA_019925795.1), and all filtered clean reads were aligned to the index (Kim et al., 2015). Meanwhile, novel genes and transcription factors were predicted using stringtie and itak, respectively (Pertea et al., 2015; Zheng et al., 2016). All genes were functionally annotated by comparing against public databases, including Nr (NCBI non-redundant protein sequences), Pfam (Protein family), KOG (euKaryotic Ortholog Groups), Swiss-Prot, GO (GeneOntology) and KEGG (Kyoto Encyclopedia of Genes and Genomes). Featurecounts was used to calculate the counts of alignment files, then the gene expression of each transcript was normalized through fragments per kb of transcript per million (FPKM) method (Mortazavi et al., 2008; Liao et al., 2014). The p value was corrected using the Benjamini & Hochberg method, and the thresholds of (adjusted p<0.05) and (|log_2_ fold change|>1) were used to judge the significance of the DEGs between two groups of samples through DEseq2 (Love et al., 2014). Clusterprofiler was applied to analyze the statistical enrichment of DEGs in the KEGG pathway and GO terms (Yu et al., 2012).

### Combined analysis of transcriptomics and volatilomics

K-means cluster analysis was performed using the FPKM values of union of all DEGs between groups of samples. In order to gain insight into links between genes and metabolites, pearson correlation coefficient were calculated between the various metabolites and the DEGs in terpenoids biosynthesis. Correlations with a coefficient absolute value > 0.975 were considered to be crucial relationships. In addition, the networks were visualized by cytoscape (Shannon et al., 2003).

### Verification of quantitative real-time PCR

12 genes involved in aroma were chosen for qRT-PCR validation. First-strand cDNA synthesis was performed using the PrimescriptRT reagent kit with a gDNA eraser (TaKaRa, Tokyo, Japan) according to the manufacturer’s instructions. qRT-PCR was accomplished using the TB Green^®^ Premix Ex Taq™ (TliRNaseHPlus, ROX plus) (TaKaRa) on an ABI 7500 system (Applied Biosystems, Foster City, CA, USA). The 18s rRNA gene was used as an internal control, and each analysis had three biological replicates. The relative expression levels were calculated using the 2^−ΔΔCt^ method (Livak and Schmittgen, 2001), and the primers sequences are shown in the **Supplementary Table 1**.

## Results

### Volatile organic compounds in *Dendrobium chrysotoxum*

**Figure 2:**
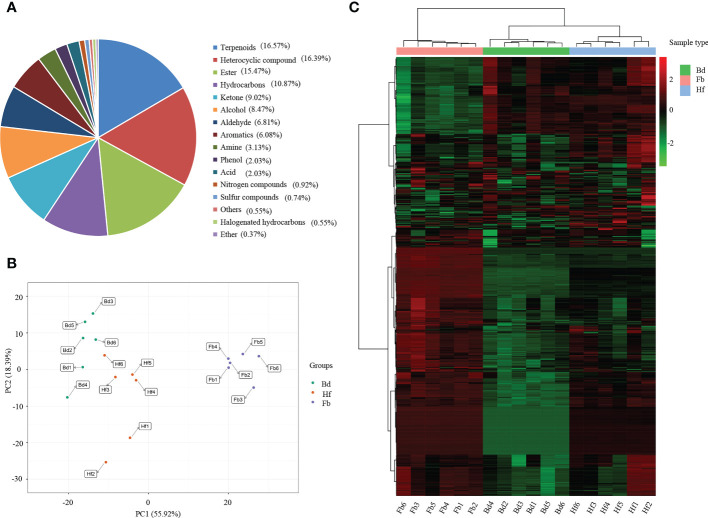
**(A)** Classification and proportion of total 543 volatile organic compounds (VOCs) detected in flowers of *D. chrysotoxum*; **(B)** Principal component analysis (PCA) among samples of *D. chrysotoxum* flowers by HS-SPME-GC/MS at different flowering stages; **(C)** Hierarchical clustering heatmap of VOCs accumulation of *D. chrysotoxum* flowers.

A total of 543 VOCs were identified in the floral organs of *D. chrysotoxum* during three flowering periods (Bd, Hf and Fb) by widely-targeted volatilomics method (**Supplementary Table 2**). The VOCs could be broadly classified into 16 categories, including terpenes, heterocyclic compounds, esters, hydrocarbons, ketones, alcohols, aldehydes, aromatics, amines, phenols, acids, nitrogen compounds, sulfur compounds, halogenated hydrocarbons, ethers and other compounds (**Figure 2A**). Among them, the categories with the highest number of VOCs were terpenes (90, 16.57%), followed by heterocyclic compounds (89, 16.39%) and esters (84, 15.47%). The three types of substances mentioned above were considered to be the main components of the floral fragrance of *D. chrysotoxum*. In addition, of the 543 VOCs, 66 were not detected in the Bd samples, and eight VOCs were unique to the Fb samples.

Principal component analysis (PCA) of the 543 VOCs showed that the distribution of Hf group was located between Bd and Fb groups, which was consistent with the flowering process of floral organs (**Figure 2B**). It was noteworthy that the distribution of Bd and Hf groups on PC1 was closer than that of the Fb group. This result indicated that the volatile metabolite compositions of Bd and Hf groups were less different, and the difference gradually increased as flowers blooming. The hierarchical clustering heatmap also showed the similar results, Bd and Hf groups were firstly clustered into one class before they clustered with Fb group (**Figure 2C**). All the above results revealed that the difference of VOCs in the floral organs of *D. chrysotoxum* gradually became larger with the development of its flowering process.

### Differential VOCs in the different stages of flowering process

**Figure 3:**
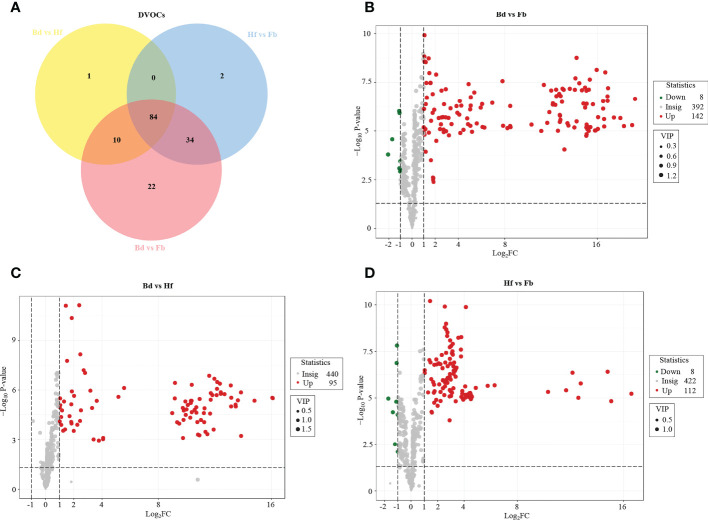
Differential volatile organic compounds (DVOCs) between different stages. **(A)**Venn diagram of DVOCs in *D. Chrysotoxum* flowers. Volcano plots of DVOCs between different pairs of groups. Bd *vs.* Fb **(B)**, Bd *vs.* Hf **(C)** and Hf *vs.* Fb **(D)**. The green dots represent down-accumulated VOCs, and the red dots represent up-accumulated VOCs between different caparisons.

**Figure 4:**
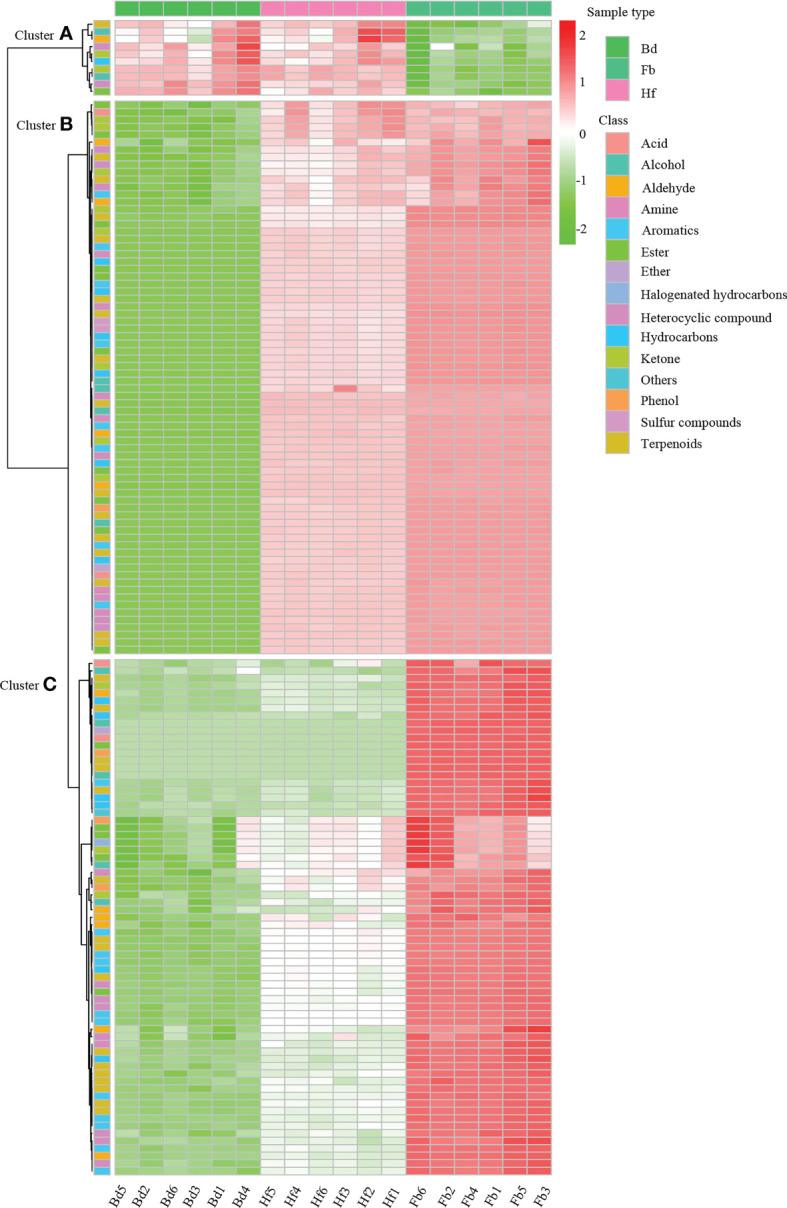
The accumulation pattern of 153 DVOCs among Bd, Hf and Fb groups in *D. chrysotoxum*. VOCs of cluster A were all significantly down-accumulated at Hf or Fb, and VOCs of cluster B and C were all significantly up-accumulated at Hf or Fb. In the graphic, red color represents up-accumulated VOCs and green color represent down-accumulated VOCs.

In order to further reveal the differences of volatile metabolites among samples of different flowering stages in *D. chrysotoxum*, OPLS-DA was performed to screen the differential VOCs between pairs of flower samples (Bd *vs*. Hf, Hf *vs*. Fb, Bd *vs*. Fb). Differential VOCs (DVOCs) were screened out between two different groups of samples under the following condition: |Log_2_ fold change|≥1.0, variable importance for projection (VIP) value > 1 and p value < 0.05. The R^2^X and Q^2^ values for each OPLS-DA models were greater than 0.95, which indicated that there were no over-fitted models, and OPLS-DA models had good discriminatory ability for samples in this study. As shown in **Figure 3A** and **Figure 4**, 153 DVOCs were screened out between different pairs of samples in total (**Supplementary Table 3**). Of which, terpenes accounted for the largest number (32, 20.92%), followed by heterocyclic compounds (21, 13.73%), aromatics (19, 12.42%) and esters (16, 10.46%). Compared to Bd group, 95 and 142 VOCs were upregulated in the Hf and Fb groups, respectively (**Figures 3C, D**). 112 VOCs were upregulated and eight VOCs were decreased in the Hf *vs*. Fb comparison (**Figure 3B**). These results once again demonstrated that VOCs gradually accumulated, and the difference of VOCs became larger during the flowers blooming of *D. chrysotoxum* (**Figure 4**).

To clarify the changes of major VOCs during the flowering process of *D. chrysotoxum*, the DVOCs which consistently upregulated were firstly analyzed. It could be seen from the Venn diagram that there were 84 VOCs consistently differentially accumulated with the flowering process, and they were all upregulated in both Bd *vs*. Hf and Hf *vs*. Fb comparison (**Figure 3A**). Among 84 VOCs, terpenes still occupied the most significant part (22, 26.19%), followed by aromatics (16, 19.05%), heterocyclic compounds (11, 13.10%) and esters (9, 10.71%). It could be observed that the order of the percentage number of these 84 VOCs species was almost identical to that of the total of 153 DVOCs. Notably, 57 of the 84 consistently upregulated DVOCs were not detected in the Bd samples, while these VOCs significantly accumulated during Hf and reached the peak at Fb (Mainly located in cluster B of **Figure 4**). As expected, these 57 VOCs which not presented in Bd samples was mainly composed of terpenes (12, 22.22%), heterocyclic compounds (8, 14.81%), esters (8, 14.81%) and aromatics (7, 12.96%). In term of fold change, differential VOCs possessing large fold change also mostly belonged to these four categories between different pairs of samples (**Table 1**). All these results above showed that terpenes, aromatics, heterocyclic compounds and esters played a pivotal role in the formation of floral scent in *D. chrysotoxum*.

**Table 1:** Top 10 DVOCs with fold change in each comparison of samples.

Index	Compounds	Class	Log_2_ (fold change)
Bd *vs.* Hf			
KMW0259	γ-Terpinene	Terpenoids	16.09
XMW0442	βhistine	Heterocyclic compound	16.06
XMW0659	Butylbenzene	Aromatics	14.79
NMW0778	(1-methylethyl)-Benzene	Aromatics	13.85
NMW0069	2,5-dimethyl-BenzenAmine	Amine	13.84
XMW0487	6-nitro-2-Picoline	Heterocyclic compound	13.62
KMW0276	1-Octanol	Alcohol	13.47
XMW1069	3-hexenyl acid Butanoic ester	Ester	13.46
D348	cis-1-methyl-2-(1-methylethenyl)-Cyclobutaneethanol	Alcohol	13.15
WMW0011	5-methyl-2-(1-methylethenyl)-Cyclohexanone	Terpenoids	13.14
Hf *vs.* Fb			
NMW0089	(R)-(+)-β-Citronellol	Terpenoids	16.58
WMW0104	Cyclooctanemethanol	Alcohol	15.07
D373	(E,E)-2,4-Heptadien-1-ol	Alcohol	14.80
XMW0766	5-methyl-2-(1-methylethylidene)-Cyclohexanone	Terpenoids	12.77
NMW0781	4-hydroxy-Butanoic Acid	Acid	12.59
KMW0359	3-ethyl-Phenol	Phenol	12.15
KMW0144	Hexanoic acid methyl ester	Ester	11.67
D76	Octanal dimethyl acetal	Ether	10.31
XMW0296	Acetic acid octyl ester	Ester	6.29
NMW0082	(-)-trans-Isopiperitenol	Terpenoids	5.77
Bd *vs.* Fb			
XMW0442	βhistine	Heterocyclic compound	19.25
KMW0259	γ-Terpinene	Terpenoids	19.00
NMW0778	(1-methylethyl)-Benzene	Aromatics	18.29
XMW0659	Butylbenzene	Aromatics	17.98
NMW0082	(-)-trans-Isopiperitenol	Terpenoids	17.42
NMW0069	2,5-dimethyl-BenzenAmine	Amine	16.92
XMW1069	3-hexenyl acid Butanoic ester	Ester	16.88
XMW0487	6-nitro-2-Picoline	Heterocyclic compound	16.87
NMW0085	4-methyl-Benzoic Acid	Acid	16.68
NMW0089	(R)-(+)-β-Citronellol	Terpenoids	16.58

Among the eight differential VOCs specific to the Fb sample, (R)-(+)-β-citronellol was the one with the largest fold changes and fragrance. Perhaps it could be a candidate biomarker for *D. chrysotoxum* in full-bloom. By contrast, there were 10 differential VOCs which were gradually downregulated with the flowering process (Mainly located in cluster A of **Figure 4**). Pinocarveol and z-3-hexenal were included, which have strong woody and grassy flavors, respectively. In addition, it was novel that a large amounts of aromatic substances, which were previously thought to be expressed only in the full-bloom stage of *D. chrysotoxum*, were detected in the Bd samples, including α-phellandrene, α-terpinene, β-ocimene, D-limonene, p-menthene, o-cymene, terpinen-4-ol, benzeneacetaldehyde, linalool and other representative components of its floral scent.

### RNA-seq analysis of floral organs at different flowering stages

To explore the differentially expressed genes (DEGs) and related pathways involved in the difference of flower fragrance between different flowering stages of *D. chrysotoxum*, nine cDNA libraries constructed using total RNA from the budding (Bd), half-open (Hf) and full-bloom (Fb) flowers were sequenced on the Illumina NovaSeq platform. A total of 411,414,802 raw reads and 399,534,816 clean reads from nine samples were obtained. The average fragments scoring Q20 and Q30 of clean reads were 97.20 and 92.25%, respectively (**Supplemental Table 4**). A total of 86.56% of the clean reads were mapped uniquely against the reference genome.

**Figure 5:**
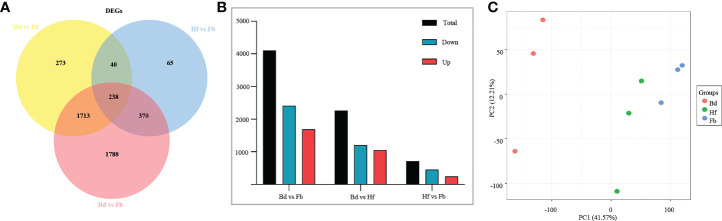
**(A)** Venn diagram of differentially expressed genes (DEGs) in *D. Chrysotoxum* flowers. **(B)** The number of DEGs between different pairs of groups. **(C)** Principal component analysis (PCA) among samples of *D. Chrysotoxum* flowers by gene expression at different flowering stages.

The fragments per kilobase of exon per million fragments mapped (FPKM) method was used to quantitate the expression level of genes, and DEGs were screened out between pairs of flower samples (Bd *vs*. Hf, Hf *vs*. Fb, Bd *vs*. Fb). The followed parameters were used as the threshold to judge the significance of the DEGs: FDR (adjusted p value) < 0.05 and |log_2_ fold change|≥1, which resulted in a set of 4,487 DEGs in total (**Figure 5A**; **Supplemental Table 5**). Nevertheless, the trends of gene expression between samples showed an opposite situation to VOCs. Compared to Bd group, 2,264 genes were differentially expressed in the Hf group. Then, only 251 DEGs were upregulated and 462 DEGs were downregulated in the Hf *vs*. Fb comparison (**Figure 5B**). It could be seen that the number of DEGs between pairs of groups was gradually decreased as the flowering process. The PCA based on FPKM values showed similar result that the biological replicates of Bd samples clustered together and were significantly separated from the two other samples (Hf and Fb) (**Figure 5C**). These results indicated that the difference of gene expression became smaller during the flowers blooming of *D. chrysotoxum*.

### Differentially expressed genes in aroma-related pathway

**Figure 6:**
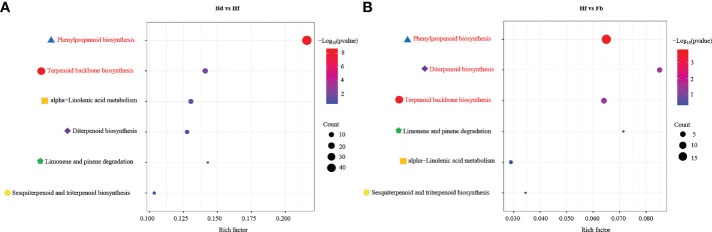
Enrichment analysis of DEGs involved in aroma-related pathway between different pairs of groups. Bd *vs.* Hf **(A)** and Hf *vs.* Fb **(B)**. Pathways with significant enrichment were highlighted by red color, and the same pathways were marked with the same color shape for comparison purposes. The rich factor was the ratio of the number of genes associated with the pathway in the DEGs to the number of genes associated with the pathway in the whole genome.

KEGG pathway enrichment analysis was performed between each comparison (Bd *vs*. Hf, Hf *vs*. Fb), in order to identify in which aroma-related metabolic pathways the DEGs were involved. The result of DEGs enrichment between each comparison showed that the aroma-related metabolic pathways mainly included terpenoids, phenylpropanoids, and aliphatics in flowers of *D. chrysotoxum*. The DEGs in the Bd *vs*. Hf groups were significantly enriched in KEGG terms terpenoid backbone biosynthesis (ko00900, rich factor: 0.14, p-value:0.02) and phenylpropanoid biosynthesis pathways (ko00940, rich factor: 0.22, p-value: 1.78E-09), these two pathways also occupied the top position in Hf *vs*. Fb comparison (ko00900, rich factor: 0.06, p-value: 0.04; ko00940, rich factor:0.06, p-value:1.54E-04). Nevertheless, compared to comparison Bd *vs*. Hf, the rich factor and significance of these two pathways were decreased in comparison Hf *vs*. Fb (**Figure 6**). It implied that the differential expression of genes in the two pathways was gradually decreasing with the flowering process. And the enrichment of the diterpenoid biosynthesis pathway (ko00904, rich factor: 0.09, p-value: 0.04), a downstream pathway of the ko00900, reached a significant level in comparison Hf *vs*. Fb. These results suggested that genes involved in the ko00900 and ko00940 might play a significant role in aroma-related biosynthesis, especially for terpenoids and phenylpropanoids.

**Figure 7:**
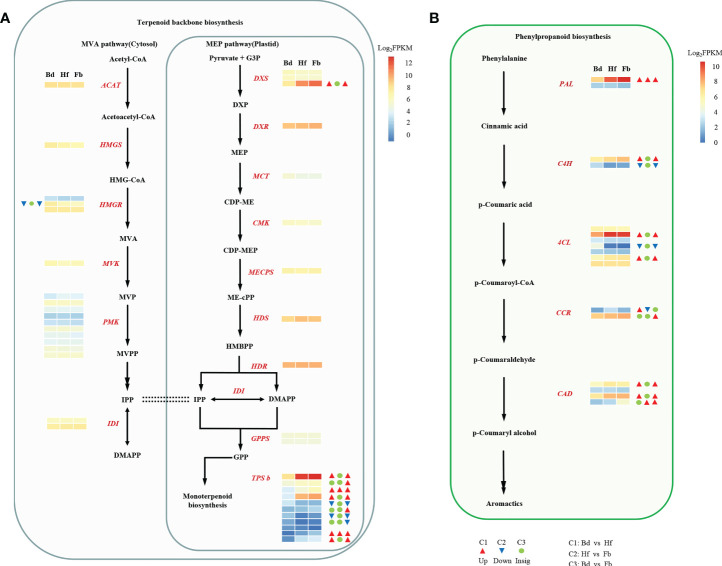
The expression profiles of genes encoding enzymes involved in terpenoids **(A)** and phenylpropanoids **(B)** biosynthetic pathway. Black bold font represented abbreviations of compounds: HMG-CoA, hydroxymethylglutaryl-CoA; MVA, mevalonate; MVP, mevalonate-5-phosphate; MVPP, mevalonate diphosphate; IPP, isopentenyl pyrophosphate; DMAPP, dimethylallyl pyrophosphate; G3P, glyceraldehyde 3-phosphate; DXP, 1-deoxy-D-xylulose 5-phosphate; MEP, methylerythritol phosphate; CDP-ME, 4-diphosphocytidyl-2-C-methyl-D-erythritol; CDP-MEP, CDP-ME 2-phosphate; ME-cPP, 2-C-methyl-D-erythritol 2,4-cyclodiphosphate; HMBPP, 4-hydroxy-3-methyl-but-2-enyl pyrophosphate; GPP, geranyl pyrophosphate. Red bold italicized font represented abbreviations of coding genes: *AACT*, acetyl-CoA acetyltransferase; *HMGS*, HMG-CoA synthase; *HMGR*, HMG-CoA reductase; *MVK*, mevalonate kinase; *PMK*, phosphomevalonate kinase; *IDI*, isopentenyl pyrophosphate isomerase; *DXS*, 1-deoxy-dxylulose-5-phosphate synthase; *DXR*, 1-deoxy-d-xylulose-5-phosphate reductoisomerase; *MCT*, 2-C-methyl-D-erythritol 4-phosphate cytidylyltransferase; *CMK*, 4-diphosphocytidyl-2-C-methyl-D-erythritol kinase; *MECPS*, 2-C-methyl-D-erythritol 2,4-cyclodiphosphate synthase; *HDS*, **(E)**-4-hydroxy-3-methylbut-2-enyl-diphosphate synthase; *HDR*, 4-hydroxy-3-methylbut-2-enyl diphosphate reductase; *GPPS*, GPP synthase; *TPS*, terpene synthase; *PAL*, phenylalanine ammonia lyase; *C4H*, cinnamate-4-hydroxylase; *4CL*, 4-coumaroyl-CoA ligase; *CCR*, cinnamoyl-CoA reductase; *CAD*, cinnamyl-alcohol dehydrogenase. Dotted line represented equivalence, double arrows represented two steps. The three squares indicated the gene expression levels (log_2_ FPKM) in **(D)** *chrysotoxum* flowers on the stage of Bd, Hf, and Fb, which were shown by a color gradient from red to white to blue. The DEGs were marked with three triangles or circles, which indicated that the differential expression in different compassion of C1 (Bd *vs.* Hf), C2 (Hf *vs.* Fb), and C3 (Bd *vs.* Fb). The red triangle denoted up-regulated, the blue inverted triangle represented down-regulated, and green circle indicated no significant difference.

The main floral volatile compounds in *D. chrysotoxum* belonged to the terpenoid and aromatics class, leading us to analyze patterns of gene expression regrading terpenoids and phenylpropanoids biosynthesis. In the pathway of terpenoid backbone biosynthesis, the expression of most genes attributed to the MEP and MVA pathways was continuous and stable with the flowering process (**Figure 7A**). With the exception of *DXS3* and *HMGR2*, whose expression were upregulated and downregulated in comparison Bd *vs*. Hf, respectively. Compared to the upstream regulatory genes, the expression of *TPSb*-like genes (responsible for the biosynthesis of terpene volatile metabolites) was more variable across flowering stages. Of the 11 *TPSb*-like genes annotated by transcriptome data, ten were differentially expressed in different comparison Hf/Fb *vs*. Bd, seven were upregulated and three were downregulated. However, in the phenylpropanoid biosynthesis pathway, the expression of its upstream regulatory genes showed a different profile (**Figure 7B**). The transcript abundance of five key structural gene families including *PAL* (1DEGs), *C4H* (1DEGs), *4CL* (2DEGs), *CCR* (1DEGs) and *CAD* (3DEGs) were higher in Fb than in Bd or Hf (**Supplemental Table 6**). Meanwhile, the expression of one *C4H* and one *4CL* genes were significantly downregulated in Hf and Fb compared with Bd.

### Combined analysis of volatilomics and RNA-seq profile

**Figure 8:**
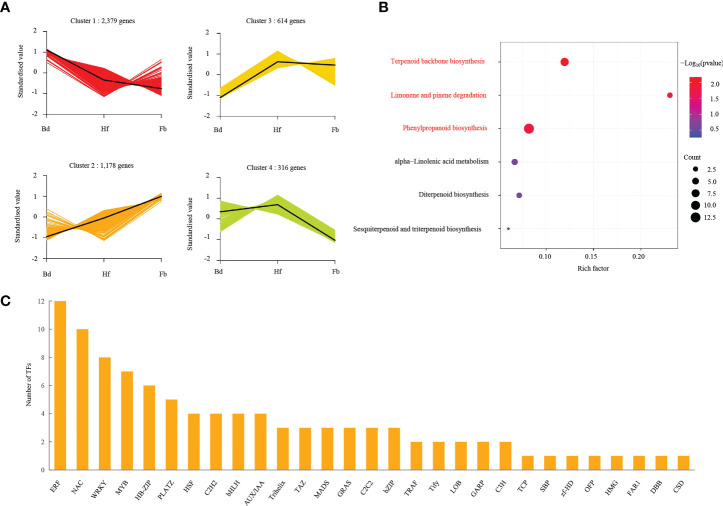
Gene regulation during flowering process. **(A)** K-means cluster analysis of co-expression gene and their expression pattern. **(B)** Aroma-related pathway enrichment analysis of DEGs in cluster 2, and pathways with significant enrichment were highlighted in red color. **(C)** The DEGs involved in transcriptome factors in cluster 2. Pathways with significant enrichment were highlighted by red color.

Terpenoids constituted the largest and most diverse class of secondary metabolites with many volatile constituents, which occupied the dominant position in the fragrance composition of *D. chrysotoxum*. To infer the underlying transcriptional regulatory relationship between terpenoids and genes, K-means cluster analysis was performed using the FPKM values of union of all 4,487 DEGs. As shown in **Figure 8A**, four clusters of DEGs could be divided during changes of flowering stages, suggesting that each cluster had a similar molecular function. Cluster one, the biggest cluster, was consisted of 2,379 genes, its expression was continuously decreased from budding stage to Full-bloom stage. And three significantly down-regulated *TPSb* genes were contained within it. Cluster two contained 1,178 genes with a pattern opposite to cluster one, and seven up-regulated *TPSb* genes were included in it. There were 614 and 316 genes were identified in cluster three and four, respectively, their expression displayed gentle trend from Bd to Fb. After screening, it could be found that the expression trend of cluster two was consistent with the accumulation of terpenoids metabolites as the flowering process. And enrichment analysis of the genes in cluster two indicated that they played a significant role in terpenoid backbone biosynthesis during flowering stage change in *D. chrysotoxum* related to some common transcription factors (TFs), such as *ERF*, *NAC*, *WRKY*, *MYB* and so on (**Figure 8B, C**; **Supplemental Table 7**).

**Figure 9:**
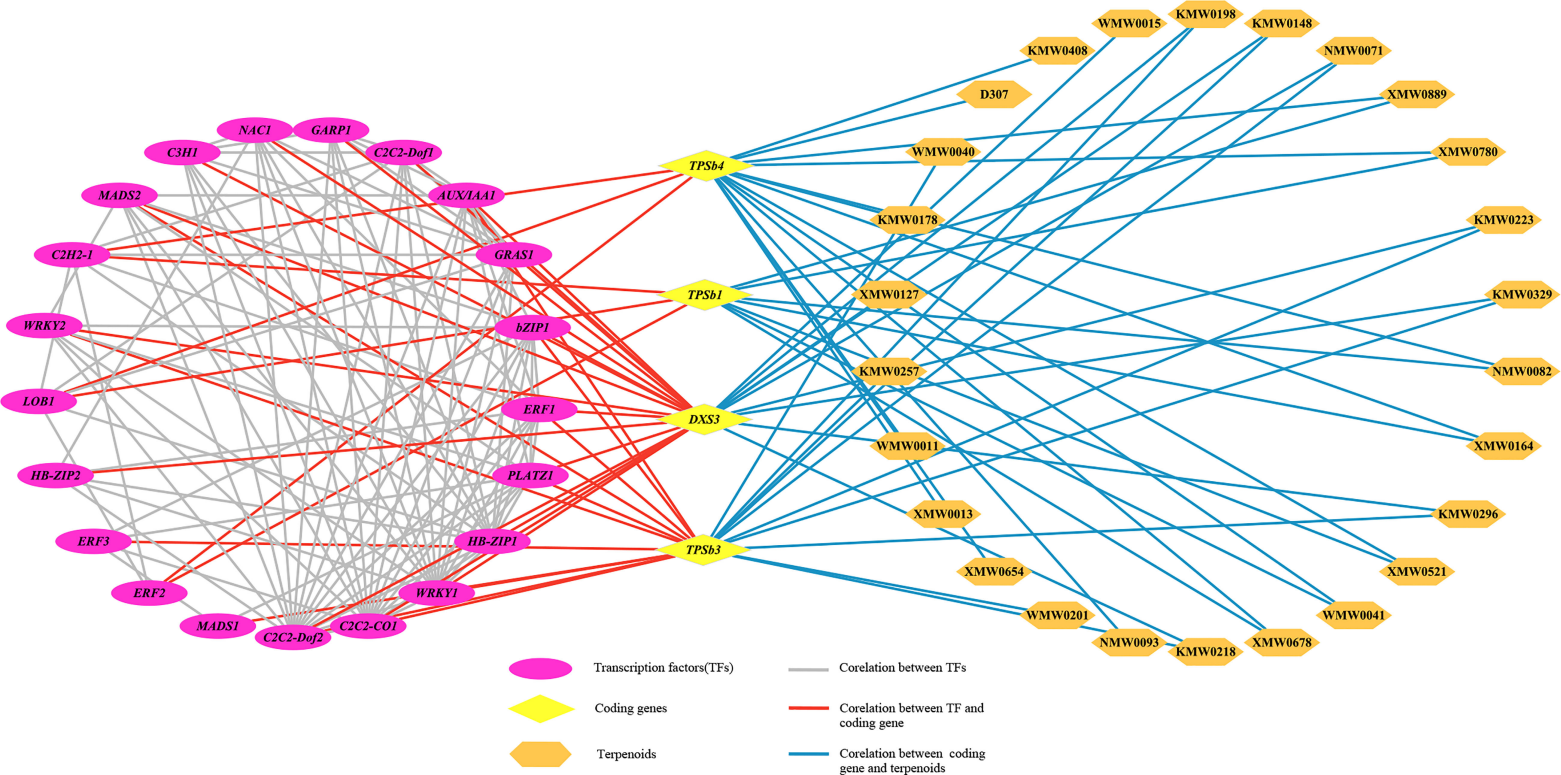
The transcriptional metabolic regulatory network consisting of terpene synthesis-related genes, candidate transcription factors and terpenoids volatile metabolites. Purple ovals represented transcription factors, yellow diamonds represented coding genes, and orange hexagons represented terpenoids. The gray line represented correlation between transcription factors, the red line represented correlation between coding gene and transcription factor, and the blue line represented Correlation line between coding gene and terpenoids.

To further reveal the correlation between terpene volatile metabolites and genes in *D. chrysotoxum* flowers, as well as to explore candidate TFs for regulating terpene biosynthesis, the correlation analysis of terpenes DVOCs and DEGs in cluster two was carried out. For correlation analysis, only correlation line with coefficients of (|r>0.975|) were retained since the DEGs in cluster two showed the same trend as the terpenes DVOCs. After a rigorous selection process, the correlation network was established to find the regulatory correlation between metabolites and genes involved in terpenes biosynthetic pathways in different flowering stages of *D. chrysotoxum* (**Figure 9**; **Supplemental Table 8**). Despite setting very severe conditions, the network still exhibited the strong correlations between the four structural genes related to terpenes synthesis pathway (*TPSb1*, *TPSb3*, *TPSb4*, *DXS3*) and 26 terpenes metabolites. *DXS3*, an upstream regulatory gene of the terpene synthesis pathway, was expectedly associated with nine aromatic terpenes and 16 TFs, respectively. Among the three TPS genes in the network, *TPSb3* was linked to the most aromatic terpenes, including α-pinene, α-phellandrene, α-terpineol, terpinene, terpinolene, trans-β-ocimene, and eucalyptol. In addition, *TPSb1* and *TPSb4* were jointly associated to the same two aromatic terpenes, theaspirane and isopinocarveol. On the other hand, some important TFs, were significantly and positively correlated with the four structural genes, including *ERF*, *MADS*, *WRKY* and so on. It indicated that these TFs might be involved in the regulation of biosynthesis of terpenoids volatile metabolites in *D. chrysotoxum*.

### Verification of gene expression

**Figure 10:**
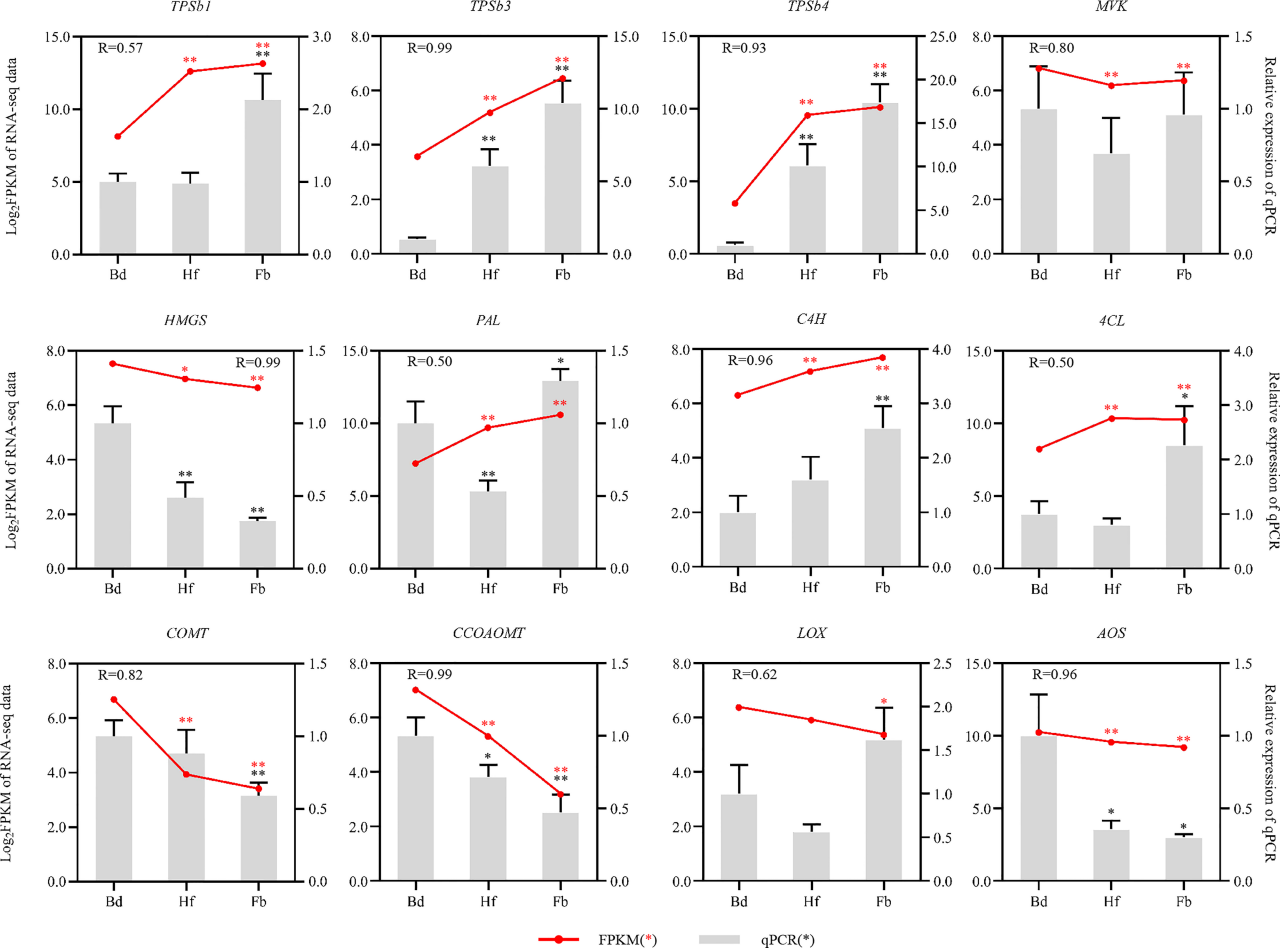
Expression patterns of 12 aroma-related genes as verified by qRT-PCR. Red dots and lines represented the FPKM value from RNA-seq data, and grey rectangle denoted the relative expression levels of genes. The red and black asterisks referred to the statistical significance of RNA-seq and qRT-PCR, respectively (Bd *vs.* Hf/Fb). One asterisk: p-value was less than 0.05; Two asterisks: p-value was less than 0.01). *COMT*, caffeic acid O-methyltransferase; *CCOAOMT*, caffeoyl-CoA O-methyltransferase; *LOX*, lipoxygenase; *AOS*, hydroperoxide dehydratase; Other abbreviations were as shown in **Figure 7**.

In order to validate the transcriptome data, 12 genes related to terpenoid, phenylpropanoid, and fatty acids biosynthesis, were selected for expression analysis in Hb, Hf and Fb by using qRT-PCR. The relative expression of most candidate genes was similar to the RNA-seq results, indicating consistency in the RNA-seq data obtained by Illumina sequence and qRT-PCR (**Figure 10**).

## Discussion

### Floral characteristics of *Dendrobium chrysotoxum*

The floral scent is an important ornamental trait that enhances the economic value of flowering plants, and the composition of floral scent varies greatly among different species (Wei et al., 2021). In the past, limited by detection techniques, researchers could only detect a few dozen kinds of volatile metabolites from the floral scent of plants. In this study, more than 500 kinds of volatile metabolites were detected in the floral scent of *D. chrysotoxum* using widely-targeted volatilomics (WTV) method (Yuan et al., 2022). A large number of volatile substances that had never been detected in dendrobium flowers before were identified, such as bergamotene, citronellol, eremophilene, naphthalene, theaspirane and ylangene. This result greatly enriched the candidate metabolites of dendrobium floral scent, and also updated our perception of floral scent. Perhaps the complexity of dendrobium floral composition was far beyond our expectation.

Terpenoids, or terpenes, were the most representative group of plant volatile metabolites, which occupied a dominant position in the floral scent components of many dendrobiums (Ramya et al., 2020). The volatile components of *D. hancockii* and *D. officinale* were mainly terpenes (Li et al., 2021). In the research, terpenoids were the most abundant class of metabolites, both in all detected metabolites and differential metabolites between different flowering stages. It indicated that terpenoids played the most significant role in the formation of floral fragrance in *D. chrysotoxum*. The emission of volatile terpenes is often temporal specific in higher flowering plants (Bouwmeester et al., 2019). Previous studies have concluded that some characteristic terpenes only could be detected in *D. chrysotoxum* at full-bloom stage, such as α-phellandrene, α-terpinene, β-ocimene, D-limonene, p-menthene, o-cymene and linalool (Huang et al., 2018). However, the experimental results of the study showed that these representative terpenes were expressed as early as the budding stage. It suggested that the formation of floral characteristics of dendrobium might begin at the budding stage rather than at the full-bloom stage.

### Volatile terpenoids metabolism genes in *D. chrysotoxum*

The existing studies on the biosynthesis of orchids fragrance were mainly focused on isolation and characterization of terpene synthase (TPS) genes encoding the key enzymes responsible for the synthesis of terpenes (Ramya et al., 2019; Ramya et al., 2020). Nevertheless, little information was referable about the biosynthesis pathways responsible for dendrobium floral scent. In order to systematically and comprehensively reveal the expression patterns of genes involved in the aroma-related pathways in dendrobium, a series of RNA-seq and referenced analysis were carried out using floral organs of *D. chrysotoxum* at different flowering stages as material.

From an overall view, the differences in gene expression became progressively smaller with the flowering process, which was the opposite of the accumulation of metabolites. This opposite trend might reflect the temporal asynchrony between upstream genes regulation and downstream metabolite biosynthesis. It indicated that the genes regulation of floral scent biosynthesis might have started at the budding stage, or even earlier in *D. chrysotoxum*, and this hypothesis also explained why some representative volatile metabolites were detected in dendrobium buds. The asynchrony was also exhibited in specific terpene biosynthetic pathways, the expression of most upstream structural genes was continuous and stable at different flowering stages, including *DXS*, *DXR*, *MCT*, *CMK*, *MECPS*, *HDS*, *HDR*, *IDI*, *ACAT*, *HMGS*, *HMGR*, *MVK*, *PMK* and other genes attributed to the MEP and MVA pathways (Dudareva et al., 2013). Their continued stable expression ensured a flow of substrates for specific terpene synthesis. In contrast, downstream *TPSb* genes were mostly showed up-regulated trend with the flowering process, which was consistent with the accumulation of terpenoids volatile metabolites in floral organs. We might venture to speculate that the different expression patterns of upstream and downstream genes in the terpene biosynthesis pathway that endowed *D. chrysotoxum* with the distinction in floral characteristics at different flowering stages, as well as ensured the strong and persistent aroma at the full-bloom stage.

### Transcriptional regulatory network for terpenoids biosynthesis in *D. chrysotoxum*

Terpene synthases were directly responsible for the formation of terpenoids diversity in plants, and a considerable amount of TPS was multi-product enzymes that could produce multiple volatiles from a single substrate (Degenhardt et al., 2009; Schilmiller et al., 2009; Shimada et al., 2014). It also explained why the 3 TPS genes in the network could be associated with multiple terpenoids. Notably, *TPSb3* was associated with some representative aromatic terpenes of dendrobium floral scent, including α-pinene, α-phellandrene and trans-β-ocimene. It suggested that TPSb3 might played a key role in the formation of dendrobium fragrance, and its function deserved further verification and study. Except for TPS genes, terpenoids biosynthesis was also regulated by several transcription factors (TFs), such as *bHLH*, *bZIP*, *ERF*, *NAC*, *MYB*, and *WRKY*. *PbbZIP4*, *PbBHLH2* and *AaNAC4* could regulate monoterpenes biosynthesis in *Phalaenopsis aphrodite*, *Phalaenopsis bellina* and *Actinidia chinensis*, respectively (Hsiao et al., 2006; An and Chan, 2012; Nieuwenhuizen et al., 2015). *CitAP2.10*, an *ERF* family transcription factor that activated the synthesis of (+)-valencene in sweet oranges (Shen et al., 2016). And homologous TFs of *WRKY* family could regulate the synthesis of (+)-δ-cadinene in cotton (Xu et al., 2004). TFs also have some influence on each other, such as *CrWRKY1* could indirectly affect monoterpenes synthesis by downregulating expression level of other three TFs (Suttipanta et al., 2011). In the present study, a total of 100 TFs belonged to 29 TF families were associated with *TPSs* as flowering process in *D. chrysotoxum*, and some known TFs for regulating terpenes biosynthesis, including *ERF*, *NAC*, and *WRKY*, occupied the top position in them.

In summary, both *TPSs* gene function and related TFs regulatory mechanisms have significance and value for further explorations, it could be said that the study on the fragrance formation was just beginning in *D. chrysotoxum*.

## Data availability statement

All sequencing raw data have been submitted to the NCBI Bioproject (accession: PRJNA864082), including 9 SRA (sequence read archive) accession numbers: SRR20726100-SRR20726108.

## Author contributions

ZC, KX, and ZD conceived and designed the study. ZD, YJ, and WW performed the experiments. ZD analyzed the data and wrote the manuscript. KX revised the manuscript. All authors contributed to the article and approved the submitted version.

## Funding

This research was funded by Guizhou Science and Technology Support Project (20201Y098) and Youth Fund of Guizhou Academy of Agricultural Sciences (202230).

## Conflict of interest

The authors declare that the research was conducted in the absence of any commercial or financial relationships that could be construed as a potential conflict of interest.

## Publisher’s note

All claims expressed in this article are solely those of the authors and do not necessarily represent those of their affiliated organizations, or those of the publisher, the editors and the reviewers. Any product that may be evaluated in this article, or claim that may be made by its manufacturer, is not guaranteed or endorsed by the publisher.
