# Supplementary material for: Molecular and metabolic insights into floral scent biosynthesis during flowering in Dendrobium chrysotoxum
Source: Front Plant Sci. 2022 Nov 28;13:1030492. doi: 10.3389/fpls.2022.1030492 (PMC9742519; doi:10.3389/fpls.2022.1030492)
Supplement: Supplementary file 1 [file DataSheet_1.pdf]

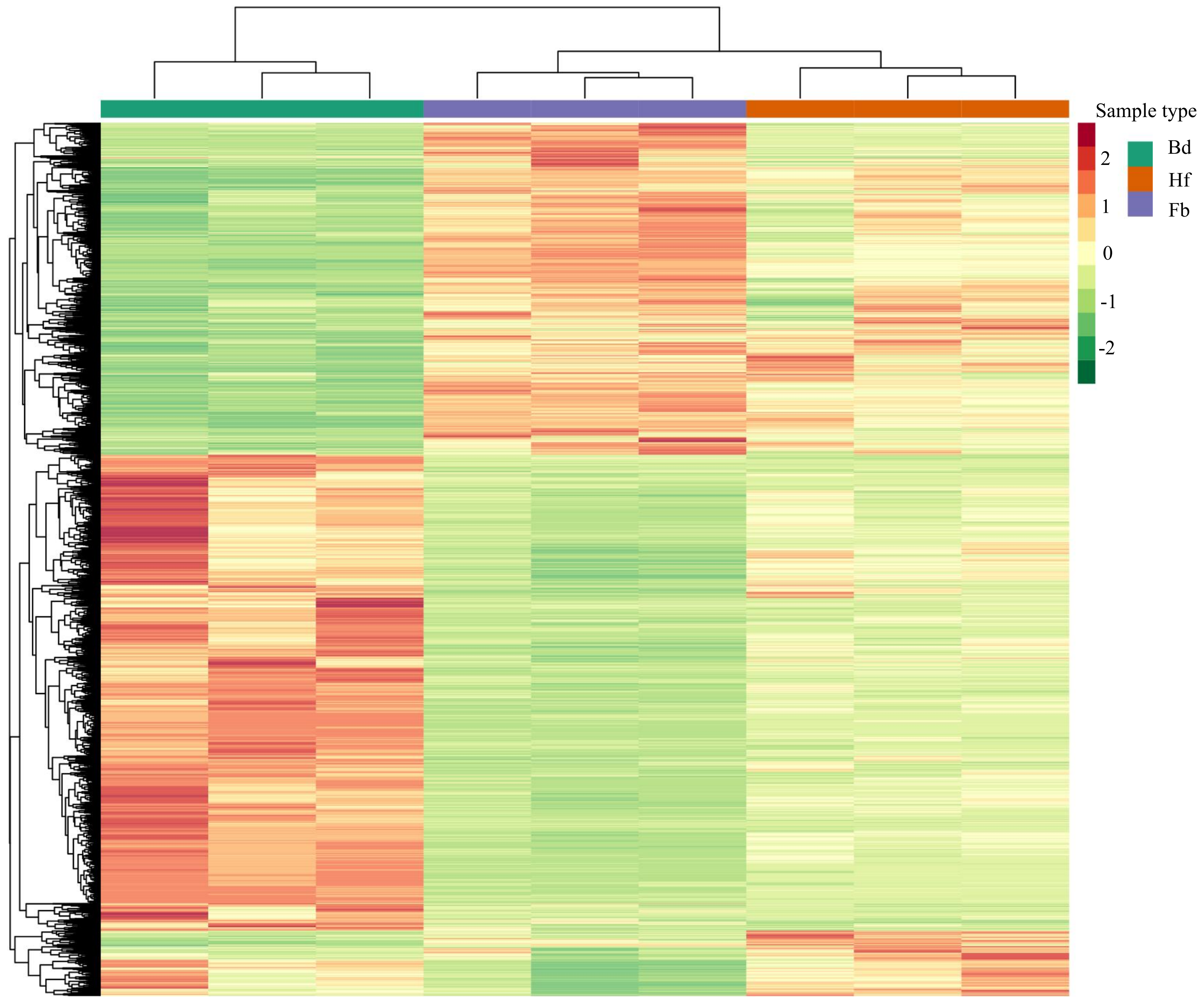

Supplementary Figure 1. Hierarchical clustering heatmap of DEGs among different pairs of comparisons in *D. chrysotoxum* flowers.
