# Supplementary material for: Molecular and metabolic insights into floral scent biosynthesis during flowering in Dendrobium chrysotoxum
Source: Front Plant Sci. 2022 Nov 28;13:1030492. doi: 10.3389/fpls.2022.1030492 (PMC9742519; doi:10.3389/fpls.2022.1030492)
Supplement: Supplementary file 2 [file DataSheet_2.pdf]

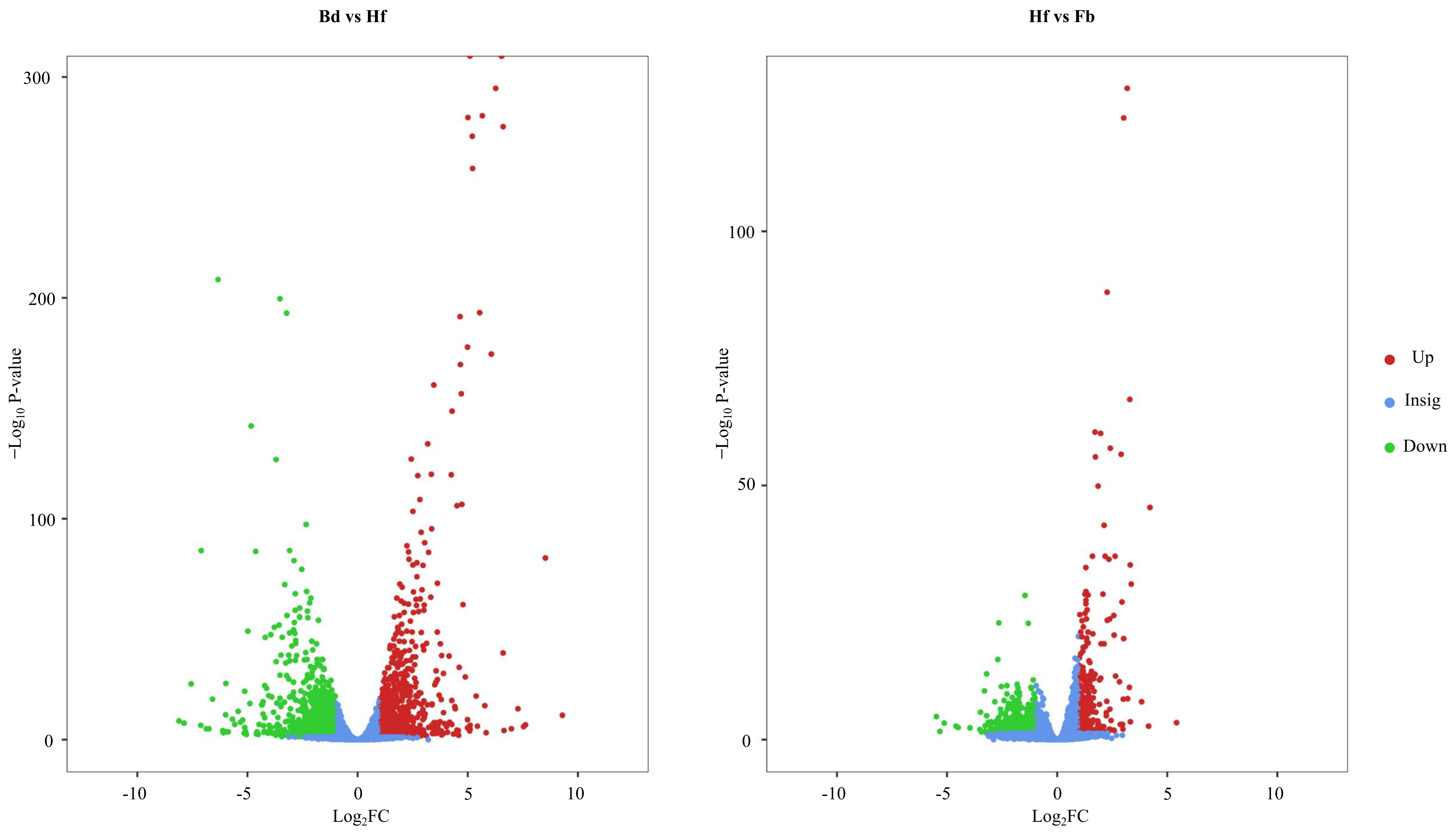


**Supplementary Figure 2**. Volcano plots of DEGs between different pairs of groups. Bd vs Hf (**A**) and Hf vs Fb (**B**). The green dots represent down-regulated DEGs, and the red dots represent up-regulated DEGs between different caparisons.
