# Supplementary material for: Molecular and metabolic insights into floral scent biosynthesis during flowering in Dendrobium chrysotoxum
Source: Front Plant Sci. 2022 Nov 28;13:1030492. doi: 10.3389/fpls.2022.1030492 (PMC9742519; doi:10.3389/fpls.2022.1030492)
Supplement: Supplementary file 3 [file DataSheet_3.pdf]

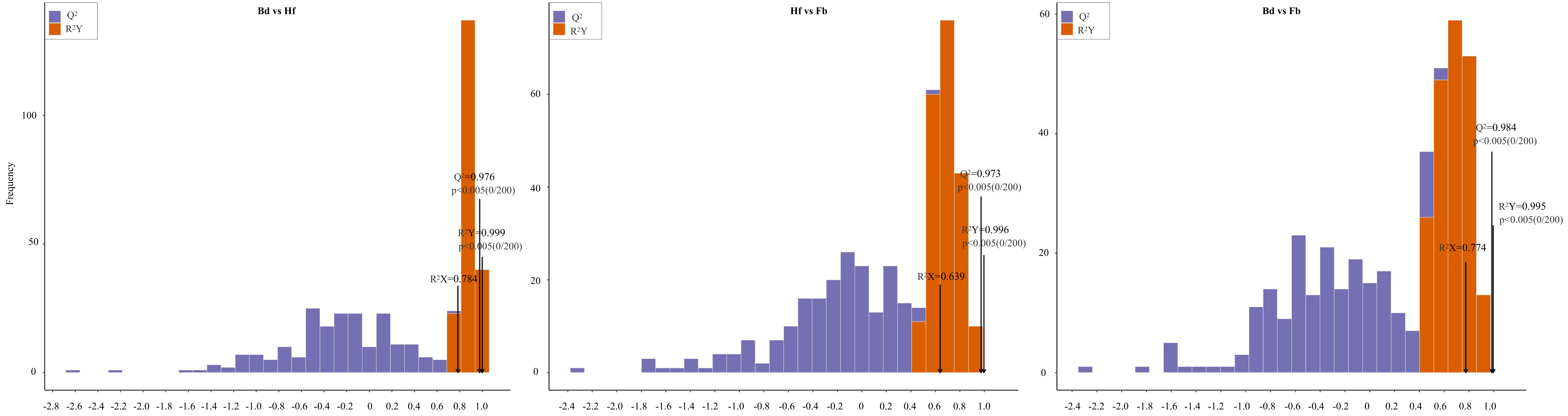

Supplementary Figure 3. Parameters and permutation test of OPLS-DA model for each group. R^2X and R^2Y denoted the explanatory rate of the model for the X and Y matrix, respectively. Q^2 represented the predictive power of the model. The closer these three indicators were to 1, the more stable and reliable the model was. The model was considered valid when it has $Q^2 > 0.5$ and excellent when $Q^2 > 0.9$. The p-value represented the probability that the result was better than the model in a random 200 permutation test.
