# Supplementary material for: Molecular and metabolic insights into floral scent biosynthesis during flowering in Dendrobium chrysotoxum
Source: Front Plant Sci. 2022 Nov 28;13:1030492. doi: 10.3389/fpls.2022.1030492 (PMC9742519; doi:10.3389/fpls.2022.1030492)
Supplement: Supplementary file 4 [file DataSheet_4.pdf]

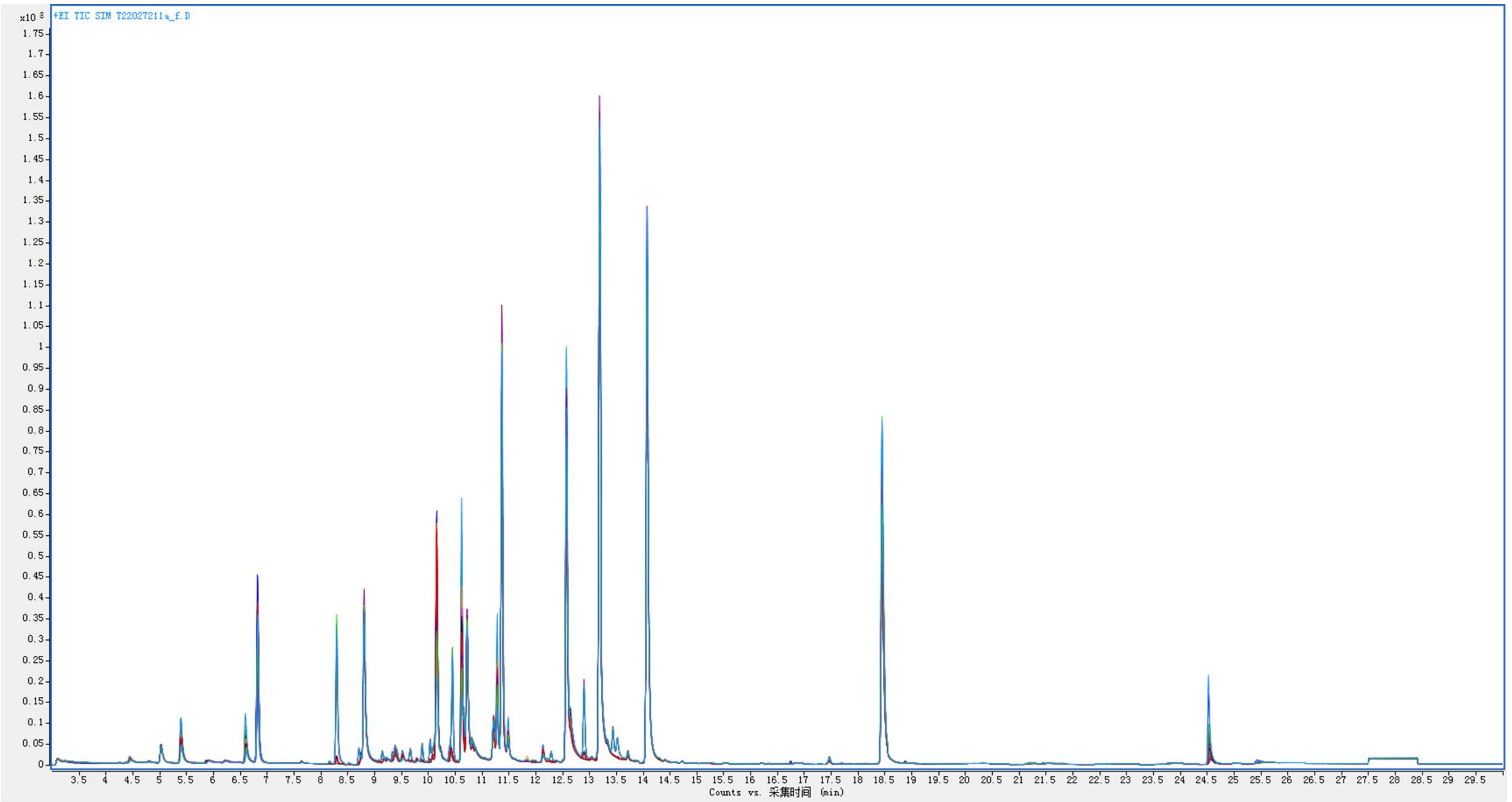

Supplementary Figure 4. TIC overlap plot of all samples of *D. chrysotoxum* flowers. TIC: total ions current, plot of the sum of the intensities of all ions in the mass spectra at each time point. The abscissa was the retention time (min) for metabolites detection, and the ordinate was the ion flow intensity (count per second) for ion detection.
